# Diagnostic Utility of NKX3.1 in Mesenchymal Chondrosarcoma on Needle Biopsy: A Case Report of a Supraclavicular Soft Tissue Mass with Literature Review

**DOI:** 10.5146/tjpath.2025.13752

**Published:** 2026-05-30

**Authors:** Sunil Pasricha, Divya Bansal, Himanshu Rohela, Anila Sharma, Rakesh Oberoi, Vikas Reddy, Ullas Batra, Anurag Mehta

**Affiliations:** Department of Pathology, Rajiv Gandhi Cancer Institute & Research Centre, DELHI, INDIA; Department of Orthopedic Oncology, Rajiv Gandhi Cancer Institute & Research Centre, DELHI, INDIA; Department of Radiology, Rajiv Gandhi Cancer Institute & Research Centre, DELHI, INDIA; Department of Medical Oncology, Rajiv Gandhi Cancer Institute & Research Centre, DELHI, INDIA

**Keywords:** Ewing sarcoma, EWSR1::NFATC2-rearranged sarcoma, Mesenchymal Chondrosarcoma, NKX3.1, Soft tissue

## Abstract

We report a case of young female in her 20s who presented with a supraclavicular soft tissue mass. Diagnostic biopsy showed a malignant round cell tumor with areas of spindling and hyalinized stroma. The utilization of an immunohistochemistry panel revealed positive results for NKX2.2 and CD99 expression. This positivity led to the consideration of a differential diagnosis of Ewing sarcoma, EWSR1::NFATC2-rearranged sarcoma, and mesenchymal chondrosarcoma for further assessment. On further immunohistochemistry with NKX3.1 and EWSR1 break-apart fluorescent in situ hybridization analysis, a diagnosis of mesenchymal chondrosarcoma was rendered which was later on confirmed with biphasic histology on excision specimen. NKX3.1 is a useful immunohistochemistry marker to resolve the differentials when dealing with undifferentiated small round cell sarcoma of bone and soft tissue, especially on a needle biopsy.

## Introduction

Mesenchymal chondrosarcoma (MC), first described by Lichtenstein and Bernstein, is a high grade malignant mesenchymal tumor involving bones and soft tissues (1). MC is a rare tumor accounting for 2-4% of all chondrosarcomas with peak incidence in 2nd-3rd decade of life without any significant sex predilection. About two-third of the cases arise in bones while the remaining cases are seen in somatic soft tissue of which meninges are the most common site (2–5).

MC have characteristic biphasic histomorphology comprising of variable mixture of well differentiated hyaline cartilage admixed with small to intermediate sized poorly differentiated round cells and hemangiopericytomatous vasculature (2–5). In diagnostic needle biopsy, the round cell tumor component is often sampled and often construed as any other malignant round cell tumor (MRCT). Ewing sarcoma (ES) and EWSR1::NFATC2-rearranged sarcoma are the most pertinent differentials of MC. With immunohistochemistry (IHC), all these three entities will show CD99 and NKX2.2 immunoexpression. Recently, NKX3.1 has been described as a useful diagnostic IHC marker for diagnosing MC and EWSR1::NFATC2-rearranged sarcoma (3,4). MC is characteristically defined by a recurrent HEY1::NCOA2 rearrangement representing an in-frame fusion at the mRNA level (2,3,5).

We present a rare case of MC of supraclavicular soft tissue region where NKX3.1 proved to be a potentially useful marker for establishing the diagnosis on needle biopsy.

## Case Report

**Figure 1:**
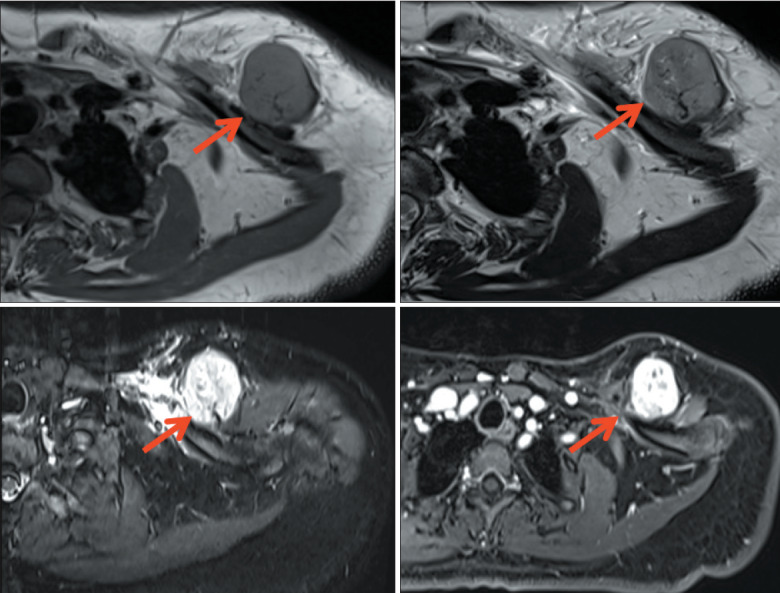
Axial T1, T2, STIR and T1+c images showing a well-defined lobulated solid mass (~3.3x2.5x3.9cm) anterior to the left clavicle, predominantly in the subcutaneous plane (arrows). It is T1 intermediate and T2/STIR hyperintense with marked enhancement on post contrast study. No evidence of bone erosion/periosteal thickening seen.

A 28-year-old female with no significant past or family history presented with gradually increasing swelling in the left supraclavicular region for 6 months with the recent onset of pain. On physical examination, no lymphadenopathy was noted. Magnetic resonance imaging upper chest revealed a well-defined lobulated solid mass, 3.9 cm in maximum dimension in the subcutaneous plane adjacent to the clavicle, with no evidence of bone erosion Figure 1.

**Figure 2:**
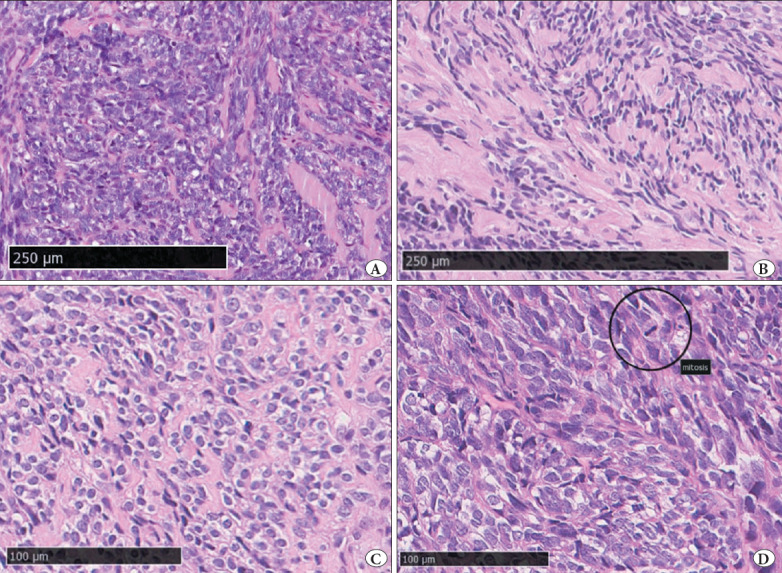
Histology of mesenchymal chondrosarcoma on small biopsy. A) Morphology of a malignant round cell tumor. B) Areas of spindled tumor cells within hyalinized stroma were noted. C) Focal areas showed stroma mimicking osteoid –like material. D) Few mitotic figures (circle) were seen in the tumor.

**Figure 3:**
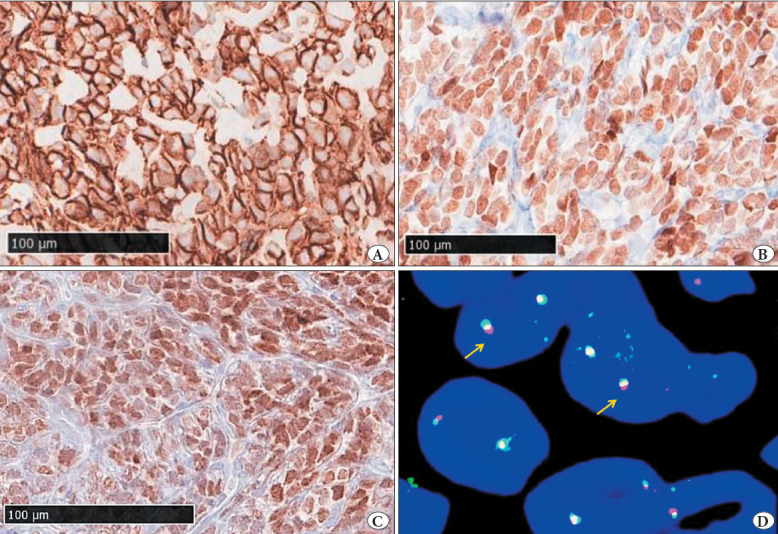
Immunohistochemistry of mesenchymal chondrosarcoma. A) Tumor cells showed strong membranous expression of CD99. B) Tumor cells showed diffuse nuclear expression of NKX2.2. C) Tumor cells showed diffuse nuclear expression of NKX3.1. D) Breakapart fluorescent in situ hybridization analysis was negative for EWSR1 gene rearrangement (arrows).

Diagnostic needle biopsy was done and histopathology examination (HPE) revealed a MRCT with focal areas showing oval to spindled cells set in a hyalinized stroma and few mitoses Figure 2. The primary panel of IHC markers revealed expression of CD99 Figure 3, NKX2.2 Figure 3 while the tumor was negative for desmin, myoD1, LCA, pancytokeratin & SSX18 which ruled out rhabdomyosarcoma, non-Hodgkin lymphomas, and poorly differentiated synovial sarcoma. In view of combined CD99, NKX2.2 positivity, the differentials considered were ES, MC, and EWSR1::NFATC2-rearranged sarcoma. NKX3.1 Figure 3 and SOX9 was done and showed diffuse nuclear positivity and ruled out classic ES. Subsequently, EWSR1 gene rearrangement was negative on break-apart fluorescent in situ hybridization (break-apart FISH) Figure 3, which ruled out EWSR1::NFATC2-rearranged sarcoma and a final diagnosis of MC was suggested. The case was discussed at a multidisciplinary tumor board meeting and excision of the mass was done thereafter.

**Figure 4:**
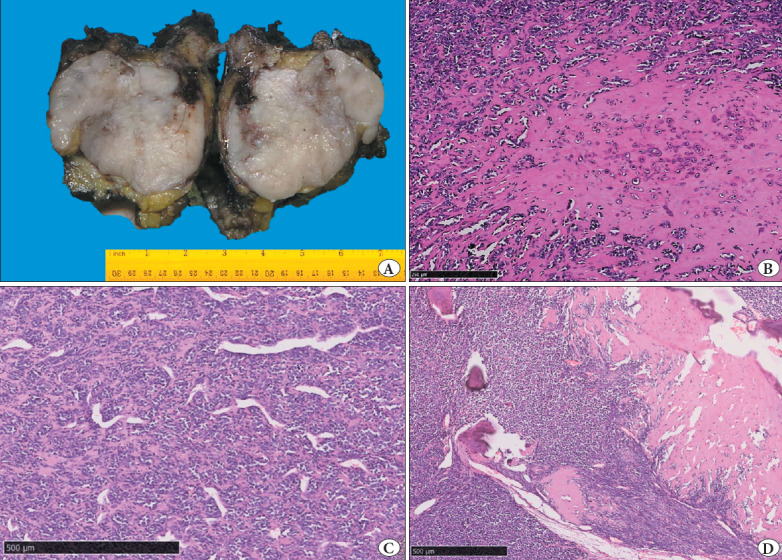
Wide local excision of mesenchymal chondrosarcoma A)Gross image showed a well circumscribed, lobulated, grey-white tumor. B) Characteristic biphasic histology of mesenchymal chondrosarcoma. C) Hemangiopericytomatous vasculature in the tumor. D) Areas of endochondral ossification in hyaline cartilage nodules.

Grossly, the tumor was relatively well circumscribed with lobulated outlines and the cut surface was homogeneous grey-white with a glistening appearance Figure 4. On HPE, the tumor was lobulated and consisted of MRCT in sheets with juxtaposed discrete area of mature hyaline cartilage Figure 4. A hemangiopericytoma-like vascular pattern was well evident and endochondral ossification was seen in occasional foci Figure 4. Hence, the final diagnosis of MC was established.

The patient was advised only adjuvant radiation therapy (60 Gy in 30 fractions) to the post-operative bed which she underwent at her native place. The patient is now on 10 months of follow-up with no evidence of disease.

## Discussion

MC is a rare and aggressive MRCT of bones and soft tissue, the diagnosis of which can be at times very challenging given its rare occurrence and especially when needle biopsy sample extracts only MRCT. Thus, the differential diagnosis covers the spectrum of MRCT in a relevant clinical and radiological context.

In the present case, the relevant differentials on needle biopsy were ES, rhabdomyosarcoma, poorly differentiated synovial sarcoma, and MC. The combined CD99 and NKX2.2 positivity prompted a diagnosis of ES. However, the presence of foci exhibiting oval to spindled blue tumor cells in hyalinised stroma provoked us to rule out MC as well. Although combined positivity of NKX2.2 and CD99 is highly sensitive and specific for the diagnosis of ES in a suitable clinical context (6,7), MC also exhibits the same immunoprofile (2,6,7).

NKX3.1 has recently emerged as a new, potentially useful marker for the diagnosis of MC (3). Syed et al. (3) evaluated NKX3.1 immunoexpression in 21 cases of MCand 32 other cases of MRCT. They found NKX3.1 positive in 66.7% of the cases (14 out 21) of MC while all other MRCT cases were negative. Glauser et al. (5) have found NKX3.1 positive expression in 35.9% of the cases (14 out of 39) of MC while all 210 cases included in the sarcoma tissue microarray were negative. Wang et al. (8) have found 100% sensitivity for NKX3.1 (positive in all 12 cases) in the diagnosis of MC. Besides MC, the utility of NKX3.1 immunopositivity has also been described in EWSR1::NFATC2-rearranged sarcoma by Yoshida et al. (4). The authors found NKX3.1 immunoexpression in 82% (9 out of 11) cases of EWSR1::NFATC2-rearranged sarcoma (diffuse and moderate to strong intensity) while 100% (all 12 cases) of MC cases were immunopositive (4). All the remaining 156 mesenchymal tumors (including 20 cases of ES) were negative for NKX3.1, except for one case of osteosarcoma which showed focal (10%) immunoexpression (4).

SOX9, like NKX3.1, was initially proposed as a reliable marker of chondrogenic differentiation in MC; however it has been shown to lack specificity, as its immunoexpression can be observed in various non-chondrogenic soft tissue tumors (9).

The HEY1::NCOA2 rearrangement is a diagnostic hallmark for MC; however, this test has limited accessibility in laboratories and should therefore be considered only in selected cases. In the present case, a combined positivity of CD99, NKX2.2 with concomitant diffuse NKX3.1 immunoexpression largely ruled out ES. However, EWSR1::NFATC2-rearranged sarcoma and MC were still differentials. Clinically, it is prudent for oncologist and pathologist to differentiate between these two entities by EWSR1 gene rearrangement by break-apart FISH. EWSR1::NFATC2-rearranged sarcoma will receive preoperative or adjuvant chemotherapy (VAC-IE regimen) while there is no cogent evidence of the benefit of adjuvant chemotherapy in MC. However, adjuvant radiation therapy is advocated. Therefore, we performed EWSR1 gene rearrangement by break-apart FISH, which was negative, and hence we suggested the final diagnosis of MC in needle biopsy.

On resection, the classical biphasic features were demonstrated on HPE Figure 4. Hence, in the present case NKX3.1 was of importance in navigation for the final diagnosis of MC in the needle biopsy consisting of only a round cell tumor component. The diagnosis of MC in diagnostic biopsy is also complicated by its enigmatic IHC profile as it may frequently show aberrant immunoexpression for desmin, myoD1, and myogenin, which may prompt an erroneous diagnosis of rhabdomyosarcoma (10,11). This further underscores the urgent need for a specific IHC marker for MC, since it is the only entity in the differentials of MRCT in which chemotherapy is not advocated as per the current therapeutic guidelines, and hence a misdiagnosis can result in unwarranted toxic effects of chemotherapy.

To conclude, NKX3.1 is a useful IHC marker to resolve the differentials when dealing with undifferentiated small round cell sarcoma of bone and soft tissue, especially in a needle biopsy that may not sample the cartilaginous component, and it has the potential to obviate the need for identifying a fusion transcript. However, a key limitation is that NKX3.1 can be expressed in EWSR1::NFATC2-rearranged sarcoma, necessitating additional molecular work-up with EWSR1 break-apart FISH, as distinguishing these two entities is critical due to their distinct therapeutic and prognostic implications.

## Conflict of Interest

The authors declare that they have no conflict of interest.

## Funding

This study was not supported by any funding.

## Ethics Approval

All procedures performed in this study involving human participants were in accordance with the ethical standards of the institutional and/or national research committee and with the 1964 Helsinki declaration and its later amendments or comparable ethical standards. The study was approved by the Institutional Review Board (Rajiv Gandhi Cancer Institute & Research Centre); vide the ethical approval letter number RES/SCM/60/2023/74

## Informed Consent

Informed consent was obtained from all individual participants included in the study.

## Consent for Publication

Consent for publication was obtained for every individual person’s data included in the study.

## Availability of Data and Material

Availability of data and material is possible upon reasonable request, deidentified for maintenance of anonymity and compliance with IRB approval.
